# Housing Stability and Neurocognitive Functioning in Homeless Adults With Mental Illness: A Subgroup Analysis of the At Home/Chez Soi Study

**DOI:** 10.3389/fpsyt.2019.00865

**Published:** 2019-11-26

**Authors:** Vicky Stergiopoulos, Adonia Naidu, Andrée Schuler, Tsegaye Bekele, Rosane Nisenbaum, Jalila Jbilou, Eric A. Latimer, Christian Schütz, Elizabeth W. Twamley, Sean B. Rourke

**Affiliations:** ^1^MAP Center for Urban Health Solutions, Li Ka Shing Knowledge Institute, St. Michael’s Hospital, Toronto, ON, Canada; ^2^Centre for Addiction and Mental Health, Toronto, ON, Canada; ^3^Department of Psychiatry, University of Toronto, Toronto, ON, Canada; ^4^Ontario HIV Treatment Network, Toronto, ON, Canada; ^5^Dalla Lana School of Public Health, University of Toronto, Toronto, ON, Canada; ^6^School of Psychology, Université de Moncton, Moncton, NB, Canada; ^7^Centre de formation médicale du Nouveau-Brunswick, Université de Sherbrooke et Université de Moncton, Moncton, NB, Canada; ^8^Department of Psychiatry, McGill University, Montreal, QC, Canada; ^9^Douglas Institute Research Centre, Montreal, QC, Canada; ^10^Department of Psychiatry, Institute of Mental Health, University of British Columbia, Vancouver, BC, Canada; ^11^Department of Psychiatry, University of California, San Diego, San Diego, CA, United States; ^12^Center of Excellence for Stress and Mental Health, VA San Diego Healthcare System, San Diego, CA, United States

**Keywords:** homelessness, mental illness, neurocognitive functioning, intervention, neurocognition, housing stability

## Abstract

**Objective:** This study examined the association of housing stability with neurocognitive outcomes of a well-characterized sample of homeless adults with mental illness over 18 months and sought to identify demographic and clinical variables associated with changes in neurocognitive functioning.

**Method:** A total of 902 participants in the At Home/Chez Soi study completed neuropsychological measures 6 and 24 months after study enrollment to assess neurocognitive functioning, specifically verbal learning and memory, cognitive flexibility, and complex processing speed. Multivariable linear regression was performed to assess the association of housing stability with changes in neurocognitive functioning between 6 and 24 months and to examine the effect of demographic and clinical variables on changes in neurocognitive functioning.

**Results:** Overall neurocognitive impairment remained high over the study period (70% at 6 months and 67% at 24 months) with a small but significant improvement in the proportion of those experiencing more severe impairment (54% vs. 49% *p* < 0.002). Housing stability was not associated with any of the neuropsychological measures or domains examined; improvement in neurocognitive functioning was associated with younger age, and bipolar affective disorder at baseline.

**Conclusions:** The high prevalence and persistence of overall neurocognitive impairment in our sample suggests targeted approaches to improve neurocognitive functioning merit consideration as part of health interventions to improve everyday functioning and outcomes for this population. Further efforts are needed to identify potential modifiable factors that contribute to improvement in cognitive functioning in homeless adults with mental illness.

## Introduction

Homelessness continues to be a significant social and health concern in North America. Individuals experiencing homelessness have higher rates of serious mental illness (SMI) and substance use than do those who are stably housed (1) and are at increased risk of chronic medical conditions (2) and premature mortality (3, 4). Furthermore, an emerging body of evidence suggests that up to 80% of people experiencing homelessness demonstrate lower than average neurocognitive functioning (5–8), including deficits in learning and memory, attention, speed of information processing, general intellectual functioning, and executive functioning (6–10). Low or impaired cognition may precede and contribute to homelessness, may be a consequence of homelessness and the poor health it entails (7, 11), or both. Cognitive impairment among people experiencing homelessness may be attributable to conditions such as mental illness, substance misuse, traumatic brain injury, and neurological and developmental disorders, among others (5, 7, 10, 11). Deficits in cognition may also arise from factors related to homelessness, including malnutrition, chronic stress, and inadequate healthcare (7). Regardless of the cause, the presence of low or impaired neurocognitive functioning among individuals experiencing homelessness and SMI has important clinical and service planning implications, as these individuals may have greater difficulties following treatment regimens, navigating the healthcare and social service systems, and participating in activities that may improve overall quality of life (12). Indeed, previous research indicates that neurocognitive functioning plays a major role in functional outcomes in this population (13, 14), as deficits in learning, memory and cognitive flexibility may impact ability to manage medications, financial matters, and negotiate complex routines and activities of daily living, including housing issues.

Prior research has also begun to examine whether neuropsychological (NP) performance may change over time as homeless individuals experience improvements in health or social circumstances, such as better nutrition, less perceived stress, and improved ability to address physical and mental health conditions. Medalia, Herlands and Baginsky (15) found that neurocognitive functioning among a small sample of formerly homeless individuals with a history of chronic mental illness, substance abuse, or both (n = 12), improved after participating in a cognitive remediation program within a supportive housing program. Seidman et al. (16) reported that after 18 months, the provision of supported housing (group homes or independent apartments) resulted in modest improvements in overall neurocognitive functioning, verbal memory, and motor speed and sequencing among homeless persons with SMI. A follow-up to this study found that gains in neurocognitive functioning after housing could be maintained after 48 months, and the presence of substance abuse might diminish the beneficial effect of housing on neurocognitive outcomes (17). These studies had several limitations, however, including moderately sized samples, and lack of a homeless comparison group.

Given the paucity of research in this area, the primary objective of this study was to examine the association between housing stability and neurocognitive functioning over 18 months in a large, well characterized sample of homeless adults with mental illness. Based on prior research, we hypothesized that housing stability would be associated with improvement in neurocognitive performance over 18 months. The secondary goal was to examine factors associated with potential changes in neurocognitive functioning in this population. An enhanced understanding of factors associated with changes in neurocognitive functioning may help identify subgroups of homeless adults that may benefit from additional interventions to improve their neurocognitive performance and optimize functional outcomes.

## Materials and Methods

This is an secondary data analysis of data collected during the At Home/Chez Soi study, an unblinded, randomized field trial examining the effect of Housing First (HF), compared to “treatment as usual” (TAU), in five cities across Canada (Vancouver, Winnipeg, Toronto, Montreal, and Moncton) [see published study protocol (18)]. The parent study was registered with the International Standard Randomized Control Trial Number Register (42520374) and was approved by the research ethics boards of all participating institutions. All participants gave written informed consent.

### Participants and Recruitment

At Home/Chez Soi eligibility criteria included legal adult status (≥18 years old, ≥19 years in Vancouver), the presence of mental disorder with or without co-existing substance use disorder, not currently being served by an assertive community treatment or intensive case management program, and being absolutely homeless (lacking regular, fixed physical shelter for at least the past seven nights) or precariously housed (e.g. living in single room occupancy, rooming house, or motel with a history of absolute homelessness in the past year). Exclusion criteria included: no legal status as a Canadian citizen, landed immigrant, refugee or refugee claimant; and relative homelessness (inhabiting spaces that did not meet the basic health and safety standards, such as living in overcrowded or hazardous conditions). Participants were enrolled in the At Home/Chez Soi study from October 2009 to July 2011. Participants were referred to the study from various community agencies that serve homeless people, including shelters, drop-in centers, inpatient programs, street outreach teams, mental health teams and criminal justice programs. In addition, researchers attended drop-in centers, shelters, and locations frequented by people who are homeless, to facilitate recruitment through self-referral.

### At Home/Chez Soi Study

At Home/Chez Soi participants were stratified at baseline into high and moderate needs groups based on their need level for mental health services (18). High-needs participants were identified as having: i) a Multnomah Community Ability Scale (19) score of 62 or lower and ii) a Mini International Neuropsychiatric Interview 6.0 (MINI 6.0) (20) diagnosis of current psychotic disorder or bipolar disorder (or an observation of psychotic behavior), and one of the following criteria: two or more hospitalizations for mental illness in any one year of the past 5 years, comorbid substance use, or recent arrest/incarceration. Moderate-needs participants were identified as all others who met eligibility criteria but were not identified as high needs. Description of the study and primary outcomes is provided in detail elsewhere (18, 21, 22). In brief, participants in the HF intervention groups received a rent supplement, along with services from either an Assertive Community Treatment team or an Intensive Case Management team, depending on their need level. Participants assigned to the TAU group had access to usual housing and mental health support services in their respective communities, which varied by site but could include access to case management or supportive housing.

### Study Measures

Neurocognitive functioning was assessed using a brief NP test battery, administered at the 6- and 24-month visits by trained research interviewers, assessing NP domains shown to be detrimentally affected among homeless adults (learning and memory, cognitive flexibility, and complex processing speed) (6). Due to the lengthy baseline interview and potential burden to participants (and consequent fatigue), NP testing was conducted at 6 and 24 months after study enrolment. Our clinical and field experience and research in this area suggest that cognitive changes are unlikely within a 6-month timeframe in the context of a housing intervention implemented over several months after study enrolment (although this is a potential factor we return to in our discussion as a limitation). Research coordinators received extensive training and supervision in the administration and scoring of the NP tests utilized. Verbal learning and memory was assessed with the Hopkins Verbal Learning Test–Revised [HVLT-R (23)]; processing speed was assessed with the Trail Making Test, Part A and B (TMT-A/B) (24) and the Digit Symbol Coding Subtest of the Wechsler Adult Intelligence Scale–Revised (WAIS-R) (25). The Trail Making Test, Part B (TMT-B) was also considered and examined as a measure of cognitive flexibility (24). The primary outcome variables were the difference between 24- and 6-month scores for the following NP measures: overall NP functioning domain *z*-score (all measures, standardized but uncorrected for age, education, and race) as well as verbal learning and memory subdomain z-score and complex processing speed and cognitive flexibility subdomain z-score (see *Statistical Analysis* section below for details).

Housing stability was assessed using the Residential Time Line Follow Back (RTLFB) inventory (26), administered by study personnel every three months. The RTLFB includes prompts and calendars to determine housing history, moves, and type of residence during a given period. Stable housing was considered as living in one’s own apartment, room, or house (or else with family) with an expected residency of at least six months or tenancy rights. Housing stability was measured as the percentage of days stably housed, calculated by dividing the total number of days spent in stable housing by the total number of days for which any type of residence data was available over the 24-month study period.

Other study measures included current mental and substance use disorders, assessed at the baseline interview using the Mini International Neuropsychiatric Interview 6.0 (MINI 6.0) (20), as well as sociodemographic characteristics, homelessness history, and comorbid medical conditions, also assessed at baseline using a series of self-reported questionnaires.

### Statistical Analysis

Of 1,326 study participants with both 6- and 24-month NP data available (participants from the Vancouver site were excluded as this site did not assess 24-month NP functioning), 902 participants were included in the analyses. To ensure data validity, participants were excluded if they were unable to complete the 6- or 24-month interviews and/or showed signs of drug or alcohol intoxication during the interview, or if they completed the interview more than 6 months past the scheduled interview date (n = 344). Participants with incomplete or missing NP data (e.g. missing data for multiple tests) were also excluded from the analyses (n = 80). We further examined whether those who were not able to complete testing, and those with missing values, differed from those who completed both assessments as per study protocol. Our analysis suggests that there were no major factors affecting the generalizability of our findings to the entire sample.

Raw scores of NP tests at 6 and 24 months were first converted into z-scores by subtracting the sample means and dividing by the standard deviation to normalize scores and enable comparisons. An overall NP *z*-score was created by averaging *z*-scores of the five NP measures (TMT-A, TMT-B, WAIS-R Digit Symbol, HVLT-R Total Recall, and HVLT-R Delayed Recall). We also examined the main neurocognitive domains or factors assessed by the five NP tests administered. To identify these factors empirically, we conducted a principal components analysis (27), and based on these findings we created two subdomain summary scores, i.e. verbal learning and memory domain (HVLT-R Total Recall and HVLT-R Delayed Recall) and complex processing speed and cognitive flexibility domain (WAIS-R Digit Symbol, TMT-A, and TMT-B). Summary *z*-scores for each domain were then generated by averaging *z*-scores of individual tests in each of the two domains. Changes in neurocognitive performance scores were calculated as the difference between 24- and 6-month scores for each outcome. Positive changes indicated neurocognitive outcomes improved at 24 months. Paired t-tests were used to compare neurocognitive performance scores at 24 and 6 months. We used ANOVA tests to compare adjusted means for summary *z*-scores between 24 and 6 months, and included a number of covariates.

To determine presence (and rate) of overall neurocognitive impairment of study participants, raw scores of NP tests were converted into demographically corrected *T*-scores using published norms for age, education, and sex (28, 29). *T*-scores were, in turn, converted into deficit scores ranging from 0 (normal), 1 (mild impairment), 2 (mild to moderate impairment), 3 (moderate impairment), 4 (moderate to severe impairment), to 5 (severe impairment) following Carey et al. (28). Deficit scores of individual tests were then averaged to calculate a Global Deficit Score (GDS). A GDS cut-off of 0.5 or greater has been validated as a cut-off point to define “overall” neurocognitive impairment and balance sensitivity and specificity (15%). This validated cut-off indicates that an individual demonstrates, on average, at least mild impairment on half of the measures of the NP test battery (28) and this cut-off mirrors the level considered to be “clinically significant” in formal NP assessments and determination of “mild” cerebral dysfunction.

Descriptive statistics were calculated for each variable of interest. Next, multivariable linear regression models examined the associations between all NP outcomes at 24-month and housing stability, age at study entry, gender (female vs. male), education (high school or higher vs. less than high school), length of homelessness (≥3 vs. < 3 years), first language (other vs. French or English), race (Indigenous, Black, Other, vs. White), study site (Moncton, Montreal, Winnipeg vs. Toronto), need level (high vs. moderate), current alcohol abuse or dependence (yes vs. no), substance abuse or dependence (yes vs. no), psychosis (psychotic disorder or mood disorder with psychotic features) (yes vs. no), major depressive disorder (yes vs. no), post-traumatic stress disorder (yes vs. no), and bipolar affective disorder at study entry (yes vs. no), adjusting for the outcome score at 6 months. All variables were chosen based on prior research (5, 7, 8, 11) and were entered into the model simultaneously. Pearson’s correlation matrix and variance inflation factors were calculated for each variable with the latter well below the generally accepted cut-off of 5.0.

We conducted additional analyses examining the changes in performance (*z*-score) on all NP outcomes for the subgroup of participants with more significant neurocognitive impairment (GDS ≥1.0), using the same regression models described above.

We used the Imputation and Variance Estimation Software IVEware version 0.2 (The University of Michigan, 2002) to conduct imputations for missing values for the TMT-B test at 24 months (n = 69, 7.6%). This software applies the sequential regression imputation method to perform imputations. We performed a single imputation (with 10 iterations) due to the low amount of missing data, using a stochastic linear regression with random perturbations to estimate TMT-B scores at 24 months with the following variables as predictors: Age, gender, education, race, language spoken at home, and NP scores at 24 months for the remaining four tests (i.e. TMT-A, HVLT-R Total Recall, HVLT-R Delayed Recall, WAIS-R Digit Symbol).

We defined statistical significance at a p value of 0.01 or less for two-tailed tests. No adjustment for multiple testing was applied (30).Statistical analyses were conducted using SAS 9.3 (SAS Institute Inc., 2011) and SPSS 20.0 (IBM Corp, 2011).

## Results

**Table 1** summarizes the sample baseline demographic and clinical characteristics. Half the sample was White (51%), one third (32%) were females, and the mean participant age at enrollment was 41.3 years (SD = 10.8). Half the sample had a lifetime duration of homelessness of more than three years (50%). Most prevalent mental disorders included major depressive disorder (55%), substance abuse or dependence (52%), and alcohol abuse or dependence (48%) (**Table 1**). The proportion of participants demonstrating overall neurocognitive impairment (using GDS ≥0.5) remained high over the study period [70% at the 6-month visit and 67% at the 24-month visit (*p* = 0.089)] (see **Table 2**). Similarly, the proportion of participants with more significant neurocognitive impairment (GDS ≥1.0) remained high over the study period although there was a modest and statistically significant decrease over time, from 54% to 49%, respectively (*p* = 0.002). Performance on two of the individual NP tests, TMT-A and TMT-B, showed statistically significant improvement over time (**Table 2**). Adjusted means for changes in each cognitive score from 6 to 24 months are presented in **Supplemental Table 1**.

**Table 1:** Baseline sample characteristics (n = 902).

	n	(%)
**Demographic variables**		
Age in years		
18–24	70	(7.8)
25–29	82	(9.1)
30–34	111	(12.3)
35–39	112	(12.4)
40–44	136	(15.1)
45–49	173	(19.2)
50–54	135	(15.0)
55+	83	(9.2)
Female	290	(32.1)
High school education or higher	442	(49.0)
First language English/French	167	(18.5)
Race		
Indigenous	193	(21.3)
White	456	(50.5)
Black	119	(13.1)
Other	134	(14.8)
Study site		
Moncton	91	(10.0)
Montreal	310	(34.3)
Winnipeg	245	(27.1)
Toronto	256	(28.3)
**Housing variables**		
Lifetime homelessness ≥3 years	454	(50.3)
**Clinical variables**		
Housing first intervention	543	(60.2)
High need	280	(31.0)
Alcohol abuse or dependence	434	(48.1)
Substance abuse or dependence	468`	(51.8)
Psychosis	389	(43.1)
Major depressive disorder	496	(54.9)
Post-traumatic stress disorder	262	(29.0)
Bipolar affective disorder	102	(11.3)
**Cognitive performance (at 6 months)**		
Overall neuropsychological functioning *z*-score, mean (SD)	0.01	(0.75)
Verbal learning and memory subdomain *z*-score, mean (SD)	0.01	(0.94)
Complex processing speed and cognitive flexibility subdomain *z*-score, mean (SD)	0.01	(0.84)

SD, standard deviation.

**Table 2:** Neuropsychological test scores at 6- and 24- month assessments (N = 902).

	6 months	24 months	*P*-value*
**Raw Scores (mean, SD)**			
HVLT-R total ^†^	19.8 (5.7)	19.9 (6.7)	0.645
HVLT-R delayed^†^	6.7 (2.8)	6.7 (2.8)	0.865
WAIS-R Digit Symbol^†^	41.7 (12.5)	41.9 (12.6)	0.574
Trail A^‡^	43.4 (20.4)	40.0 (17.6)	**<0.001**
Trail B ^‡§^	109.3 (58.5)	100.3 (50.0)	**<0.001**
***T*-Scores (mean, SD)**			
HVLT-R total ^†^	35.0 (10.8)	35.2 (11.5)	0.551
HVLT-R delayed^†^	37.5 (11.4)	37.6 (11.4)	0.675
WAIS-R Digit Symbol^†^	43.7 (10.0)	44.2 (10.0)	0.018
Trail A^‡^	39.8 (12.0)	42.4 (11.8)	**<0.001**
Trail B ^‡§^	42.0 (11.6)	44.0 (11.9)	**<0.001**
**Global Deficit Scores**			
Score (median, IQR)	1.0 (0.4–1.8)	0.8 (0.4–1.6)	
Any impairment ≥0.5 (%)	628 (69.6%)	602 (66.7%)	0.089
Severe impairment ≥1.0(%)	483 (53.6%)	438 (48.9%)	**0.002**

HVLT-R, Hopkins Verbal Learning Test-Revised; SD, Standard Deviation; WAIS-R, Wechsler Adult Intelligence Scale–Revised.

*Paired t tests (2-tailed).

^†^Number of correct responses.

^‡^Time in seconds.

^§^Missing values at 24 months (n = 69) were replaced with imputed values.

Bold-faced p-values indicate statistical significance at the 0.01 level.

### Multivariable Regression Analyses

Housing stability was not associated with any of the neurocognitive outcomes examined (**Table 3**). Older age was associated with decreases in the overall neurocognitive functioning domain [β = −0.008 (95% CI, −0.011 to −0.004)] and the complex processing speed and cognitive flexibility subdomain [β = −0.011 (95% CI, −0.015 to −0.008)]. Participants who met criteria for bipolar disorder at baseline showed increases in the verbal learning and memory subdomain [β = 0.226 (95% CI, 0.072–0.379)] compared to those who did not meet criteria for this disorder.

**Table 3:** Multivariable linear regression analyses using change in *z*-scores from 24 months to 6 months as dependent variables, adjusting for *z*-scores at 6 months (n = 902).

Predictor variable	Overall neurocognitive functioning				Verbal learning and memory domain*				Complex processing speed and cognitive flexibility domain ^†‡^			
	*?*	(95% CI)		*p*	*β*	(95% CI)		*p*	*β*	(95% CI)		*p*
**Housing variables**												
Stable housing (% days)	0.015	−0.071	0.100	0.739	0.081	−0.049	0.211	0.219	−0.016	−0.112	0.080	0.740
Lifetime homelessness (≥3 years)	0.052	−0.011	0.116	0.108	−0.002	−0.099	0.094	0.960	0.088	0.016	0.160	0.016
**Baseline demographic variables**												
Age (years)	−0.008	−0.011	−0.004	**<0.001**	−0.004	−0.008	0.001	0.152	−0.011	−0.015	−0.008	**<0.001**
Gender (female)	0.046	−0.023	0.116	0.191	0.106	0.001	0.212	0.048	0.027	−0.051	0.104	0.499
Education (≥HS)	0.063	−0.002	0.127	0.056	0.063	−0.035	0.160	0.206	0.085	0.013	0.157	0.021
First language (other)	−0.059	−0.146	0.028	0.186	−0.084	−0.215	0.048	0.213	−0.047	−0.145	0.051	0.344
Race (ref: white)												
Indigenous	0.011	−0.102	0.125	0.846	0.074	−0.099	0.247	0.403	−0.073	−0.200	0.054	0.259
Black	−0.123	−0.236	−0.009	0.035	−0.123	−0.295	0.048	0.159	−0.160	−0.288	−0.033	0.014
Other	0.001	−0.099	0.099	1.000	−0.010	−0.161	0.141	0.898	−0.026	−0.137	0.085	0.645
Study site (ref: Toronto)												
Moncton	0.090	−0.036	0.216	0.160	0.230	0.038	0.421	0.019	−0.003	−0.145	0.138	0.965
Montreal	0.107	0.017	0.198	0.020	0.267	0.13	0.404	**<0.001**	0.007	−0.095	0.109	0.895
Winnipeg	0.199	0.084	0.315	**0.001**	0.476	0.301	0.652	**<0.001**	0.035	−0.094	0.164	0.594
**Baseline clinical variables**												
High need	−0.065	−0.141	0.011	0.094	−0.093	−0.208	0.022	0.112	−0.064	−0.150	0.021	0.140
Alcohol abuse^§^	0.015	−0.055	0.085	0.678	−0.047	−0.154	0.059	0.384	0.056	−0.023	0.134	0.165
Substance abuse^§^	−0.005	−0.074	0.064	0.889	0.013	−0.091	0.118	0.802	−0.019	−0.097	0.059	0.630
Psychosis	−0.021	−0.093	0.050	0.557	−0.008	−0.117	0.1003	0.879	−0.040	−0.12	0.040	0.330
Major depressive disorder	−0.008	−0.078	0.062	0.813	−0.049	−0.155	0.057	0.365	0.027	−0.052	0.105	0.507
PTSD	0.088	0.014	0.162	0.020	0.124	0.011	0.236	0.031	0.064	−0.019	0.147	0.131
Bipolar affective disorder	0.086	−0.015	0.187	0.096	0.226	0.072	0.379	**0.004**	−0.003	−0.117	0.110	0.956
**Neurocognitive performance at 6 months**												
NP overall/domain *z*-score	−0.286	−0.331	−0.242	**<0.001**	−0.436	−0.488	−0.384	**<0.001**	−0.282	−0.327	−0.236	**<0.001**
Intercept	0.151	−0.039	0.341	0.120	−0.186	−0.472	0.100	0.202	0.415	0.201	0.629	**<0.001**
*R*^2^				0.192				0.293				0.161
Adjusted *R*^2^				0.173				0.277				0.142
RMSE				0.467				0.708				0.523
*F*-statistic	*F* (20, 902) = 10.43, *p* < 0.001				*F* (20, 902) = 18.22, *p* < 0.001				*F* (20, 902) = 8.47, *p* < 0.001			

*Average of HVLT-R total recall and HVLT-R delayed recall.

^†^Average of WAIS-R Digit Symbol, Trail A and Trail B.

^‡^Missing values at 24-month were replaced with imputed values for 69 individuals.

^§^or dependence.

HS, high school; NP, neuropsychological; RMSE, root mean squared error; PTSD, Post-traumatic stress disorder.

Bold-faced p-values indicate statistical significance at the 0.01 level.

Subgroup analyses among participants experiencing more significant neurocognitive impairment (GDS ≥1.0) yielded virtually the same results as the main findings (data not shown).

## Discussion

This is the largest study to date to document the rate of neurocognitive impairment in a well characterized sample of homeless adults with mental illness, and examine the association of housing stability with neurocognitive performance, and factors associated with changes in neurocognitive functioning over 18 months. The prevalence of neurocognitive impairment, based on the GDS, was high (70%) and remained high (67%) in our sample over the study period, with small but significant decreases in the proportion of those experiencing more severe impairment. The rates of overall neurocognitive impairment, based on the GDS, were comparable to those reported in prior studies of homeless populations (28%–82%) (6, 7), and much higher than what has been reported in the general population (16%) (29). Performance on two individual NP tests, TMT-A and TMT-B (aspects of complex processing speed and cognitive flexibility), improved significantly over time; however, these improvements, while statistically significant, did not appear to be clinically significant.

Contrary to expectations, housing stability was not associated with significant changes in the NP domains examined, namely verbal learning and memory, processing speed, and cognitive flexibility or in the overall level of neurocognitive performance. There are several possible reasons for the lack of an observed effect in our study sample. First, it is possible that neurocognitive impairment (overall and in specific domains) is enduring, for a variety of reasons related to the complex medical/psychiatric morbidities substance use disorders, and traumatic brain injury, common in this population (31). Second, changes in neurocognitive functioning in this population may require longer time periods than 18 months, our study’s follow up period, and may require active and sustained cognitive activation interventions (31, 32) and possibly physical exercise (33) to increase the likelihood of neuronal activation and growth that will eventually lead to improvements in neurocognitive status. Third, it is possible that our study was not powered to detect small changes in neurocognitive performance. Fourth, it is possible that housing stability may not sufficiently affect lifestyle, medical status, or other specific risk constellations necessary to see changes in neurocognitive functioning.

Prior research suggesting that assignment to a housing intervention improved neurocognitive functioning in a sample of individuals experiencing homelessness and mental illness lacked a control group of participants (16, 17). Neither HF nor usual services specifically targeted neurocognitive functioning for improvement. It may be beneficial to enhance housing interventions for this population to better accommodate and support those demonstrating significant neurocognitive impairment. Specifically, the integration of cognitive remediation should be considered in such interventions, as a substantial body of literature supports the use of this approach in improving neurocognitive functioning among individuals with SMI (34), including homeless individuals (15).

In terms of demographic variables, we found that older age was associated with decreases in various NP domains at 24 months, consistent with previous cross-sectional research in a similar population (12), as well as longitudinal research that found older adults to be more likely to experience decreases in neurocognitive functioning over time (35). All of the NP tests used in this study have significant age effects, and further efforts are needed to explore if changes observed in our study reflect “accelerated” or premature” aging (36, 37). Other socio-demographic factors typically associated with neurocognitive functioning, such as gender and education (38–40), did not reach statistical significance in our study. Differences were also found for study sites, reflecting variation in the regional health and social service delivery contexts and unique subpopulations of homeless adults at each study site.

In terms of baseline clinical factors, we found that the presence of bipolar disorder at baseline was associated with statistically significant improvements in neurocognitive functioning. While bipolar disorder is typically associated with impairment across a variety of NP domains (41–43), improvements in manic symptoms (44) have been associated with improvements in neurocognitive functioning. It is therefore possible that individuals with bipolar disorder experienced clinical improvement over the course of the study, explaining improvements in neurocognitive functioning. On the other hand, the presence of major depressive disorder at baseline was not associated with changes in any of the NP domains examined, despite the episodic nature of major depressive disorder and expected clinical improvement over time. Consistent with prior work (16), the presence of a substance use disorder was not related to any of the neurocognitive outcomes examined in the study. Given previous findings that substance use may moderate the effect of housing on neurocognitive outcomes (17), further research is needed to clarify the relation between substance use and cognition in homeless populations.

Several limitations to our study must be noted. First, although we administered a NP assessment battery that assessed various NP domains, it is possible that other NP domains (e.g. complex attention, visuospatial memory, or other aspects of executive functioning not captured by the TMT) may be more sensitive to changes in housing stability. Secondly, a longer treatment interval (i.e. longer than 18 months) and larger sample size may be necessary to demonstrate any effects of housing stability on neurocognitive performance. Third, beyond the baseline visit, we did not administer either a diagnostic interview such as the MINI, or standardized measures of disease specific symptom severity to examine possible changes in mental disorders during the follow-up period. Fourth, initial NP assessment took place at the 6-month study visit after study entry, raising the possibility that some improvements had already taken place during the first 6 months after study enrolment for some participants. Of note, independent sample t-test analyses comparing 6-month neurocognitive performance between the intervention and TAU groups revealed no significant group differences, and housing stability was achieved many months after study enrolment for most participants. Fifth, it is possible that the exclusion of participants with missing or incomplete NP data may have resulted in biases, and the exclusion of individuals experiencing relative homelessness suggests that findings cannot be generalized to this marginalized population. Finally, we did not examine the possibility of a moderating effect of medical status on housing stability and changes in neurocognitive functioning, which will be the focus of future research.

In summary, findings from the present study suggest that housing stability is not associated with changes in neurocognitive functioning among adults experiencing homelessness and mental illness over an 18-month time period. Additional interventions targeting neurocognitive performance, such as cognitive remediation and physical exercise may need to be considered within supportive housing programs and services. Future research should explore the multiple pathways to and distinct profiles of neurocognitive impairment in this population, and examine both longitudinal changes in neurocognition as well as their underlying neurobiological substrates to identify potential targets for intervention.

## Data Availability Statement

Anonymized At Home/Chez Soi participant data, study protocol, and statistical analysis plan will be available to investigators for studies that have received approval from independent research committees or research ethics boards. Study proposals and data access requests should be sent to the corresponding author.

## Ethics Statement

The At Home/Chez Soi study was reviewed and approved by Research Ethics Boards at each participating site. All study participants provided written informed consent to participate in the study.

## Author Contributions

VS, SR, EL, RN, and CS conceived and designed the study. VS and AN drafted the manuscript and VS supervised the overall study. RN participated in data curation and supervised the data analysis. TB participated in data curation and performed the data analysis. VS, AN, AS, TB, RN, JJ, EL, CS, EW, and SB interpreted the data and contributed to manuscript revisions and edits. All authors have approved the final manuscript.

## Conflict of Interest

The authors declare that the research was conducted in the absence of any commercial or financial relationships that could be construed as a potential conflict of interest.
