# Supplementary material for: Housing Stability and Neurocognitive Functioning in Homeless Adults With Mental Illness: A Subgroup Analysis of the At Home/Chez Soi Study
Source: Front Psychiatry. 2019 Nov 26;10:865. doi: 10.3389/fpsyt.2019.00865 (PMC6889850; doi:10.3389/fpsyt.2019.00865)
Supplement: Supplementary file 1 [file Table_1.pdf]

Supplemental Table I. Adjusted means for changes in cognitive domain scores between 6 and 24 months

	Overall neuropsychological functioning z-score			Verbal learning and memory domain z-score[¶]			Complex processing speed and cognitive flexibility domain z-score[§]		
	mean	SD	p*	mean	SD	p*	mean	SD	p*
**Stable Housing (% days)**			0.542			0.139			0.329
<25%	0.028	0.511		0.005	0.837		0.044	0.570	
25-50%	0.027	0.522		0.156	0.716		-0.060	0.604	
>50%	-0.013	0.513		-0.026	0.847		-0.004	0.557	
**Lifetime homelessness (≥ 3 years)**			0.189			0.612			0.136
No	-0.023	0.494		-0.014	0.813		-0.028	0.544	
Yes	0.022	0.532		0.014	0.853		0.028	0.584	
**Age at 6-month testing**			0.267			0.529			0.114
<25	0.020	0.505		-0.132	0.926		0.120	0.584	
25-29	0.007	0.588		-0.012	0.879		0.019	0.695	
30-34	0.032	0.550		0.105	0.958		-0.016	0.578	
35-39	0.043	0.532		0.058	0.895		0.032	0.537	
40-44	0.045	0.475		0.051	0.819		0.040	0.545	
45-49	0.014	0.477		-0.002	0.755		0.025	0.501	
50-54	-0.097	0.514		-0.081	0.778		-0.108	0.578	
>=55	-0.069	0.491		-0.044	0.695		-0.086	0.548	
**Gender**			0.627			0.341			0.841
Male	-0.006	0.514		-0.018	0.835		0.003	0.573	
Female	0.012	0.513		0.038	0.829		-0.005	0.549	
**Education (>=HS)**			0.130			0.045			0.744
No	0.025	0.504		0.054	0.858		0.006	0.554	
Yes	-0.026	0.522		-0.057	0.803		-0.006	0.578	
**First language**			0.908			0.692			0.831
English/French	0.001	0.512		0.005	0.845		-0.002	0.563	
Other	-0.004	0.520		-0.023	0.779		0.008	0.574	

**Race**			**<0.001**			**<0.001**			0.855
Aboriginal	0.140	0.550		0.323	0.945		0.018	0.545	
Black	-0.103	0.528		-0.199	0.706		-0.039	0.721	
Other	-0.008	0.441		-0.031	0.695		0.007	0.540	
White	-0.030	0.503		-0.075	0.816		0.000	0.535	
**Site**			**<0.001**			**<0.001**			0.695
Moncton	-0.026	0.478		-0.010	0.809		-0.037	0.545	
Montreal	-0.007	0.463		-0.037	0.749		0.013	0.510	
Winnipeg	0.129	0.554		0.288	0.900		0.023	0.570	
Toronto	-0.105	0.520		-0.227	0.795		-0.025	0.629	
**Needs level**			0.523			0.641			0.612
Moderate	-0.007	0.515		-0.009	0.840		-0.006	0.561	
High	0.016	0.510		0.019	0.818		0.014	0.576	
**Alcohol abuse**			0.066			0.275			0.087
No	-0.030	0.478		-0.029	0.718		-0.031	0.559	
Yes	0.033	0.547		0.031	0.941		0.033	0.570	
**Substance abuse**			0.664			0.468			0.956
No	-0.008	0.529		-0.021	0.784		0.001	0.591	
Yes	0.007	0.499		0.019	0.876		-0.001	0.540	
**Psychosis**			0.913			0.814			0.948
No	-0.002	0.525		-0.006	0.885		0.001	0.555	
Yes	0.002	0.498		0.007	0.760		-0.001	0.578	
**Major depressive disorder**			0.655			0.571			0.910
No	-0.008	0.480		-0.017	0.758		-0.002	0.571	
Yes	0.007	0.539		0.014	0.890		0.002	0.561	
**PTSD**			**0.002**			**0.001**			0.163
No	-0.033	0.499		-0.058	0.810		-0.017	0.554	
Yes	0.082	0.539		0.143	0.872		0.041	0.590	
**Bipolar affective disorder**			0.155			**0.005**			0.553
No	-0.009	0.502		-0.028	0.803		0.004	0.566	
Yes	0.068	0.592		0.217	1.018		-0.031	0.563	

PTSD, Post-Traumatic Stress Disorder; SD, Standard Deviation
¶ Average of HVLT-R Total recall and HVLT-R delayed recall
§ Average of WAIS-R Digit symbol, Trail A and Trail B
* ANOVA/T-Test comparing change in Z-scores between 6 and 24 months
